# A Novel Approach for the Treatment of Pulmonary Artery Aneurysm Repair Using Inclusion Technique: A Case Report

**DOI:** 10.7759/cureus.36456

**Published:** 2023-03-21

**Authors:** Mariana Flaifel, Rohan Suresh Daniel, Hayato Nakanishi, Christian A Than, George Shiakos, Ioannis Tzanavaros

**Affiliations:** 1 Cardiothoracic Surgery, St George's University of London, London, GBR; 2 Cardiothoracic Surgery, University of Nicosia Medical School, Nicosia, CYP; 3 Cardiothoracic Surgery, International Journal of Clinical Research Central, Delaware, USA; 4 Cardiothoracic Surgery, The University of Queensland, Brisbane, AUS; 5 Cardiothoracic Surgery, Cardiac Innovation Center of Apollonion Private Hospital, Nicosia, CYP

**Keywords:** graft inclusion, aneurysmectomy, cardiac surgery, aneurysm, pulmonary artery

## Abstract

Pulmonary artery aneurysm (PAA) is a rare disease with life-threatening complications, especially when accompanied by pulmonary artery hypertension. Due to its rarity, there are currently no specific guidelines for the treatment of PAA. Several surgical techniques have been described to be beneficial in the treatment of PAA originating at the pulmonary trunk. However, several adverse complications have been described for traditional techniques. In this case, we present the first successful repair of PAA with idiopathic pulmonary artery hypertension using a graft inclusion technique.

## Introduction

Pulmonary artery aneurysm (PAA) is a rare disease characterized by focal dilation of the pulmonary artery (PA) involving all three layers of the vessel wall. The condition is diagnosed when the diameter of the vessel dilation is greater than 1.5 times the normal upper limits [1] or when the pulmonary trunk measures greater than 4 cm [2]. PAA has an incidence of approximately one in 14,000 postmortem examinations, making it a rare condition [3]. The etiology of PAA can be attributed to various congenital and acquired etiologies, including congenital heart defects, connective tissue abnormalities, and pulmonary hypertension (PAH) [4]. PAH can be defined as a mean PA pressure ≥ 20 mmHg at rest [2] and is an important contributor to PAA formation, and has been linked as a clinical manifestation of its presence [5].

The presence of PAA and PAH concomitantly increases the risk of vessel wall rupture and dissection of the aneurysm [6]. In addition, PAA poses a risk of compressing neighbouring structures such as the left main coronary artery, the main bronchus, and/or the recurrent laryngeal nerve [6]. Among these life-threatening complications, a ruptured PAA can result in high mortality rates between 50-100% [7]. To prevent potentially fatal complications associated with PAA, early surgical intervention may be warranted in selected cases where patients present with both PAA and PAH. However, due to the low prevalence of PAA and the heterogenous contributing etiologies, no clear guidelines for treating PAAs have been established [8].

A variety of surgical procedures have been proposed for the treatment of PAA. This case report details a novel surgical approach using an inclusion technique performed on a patient with a 6.1 cm diameter PAA and concomitant idiopathic PAH (IPAH). This technique has been used successfully in the treatment of other types of aneurysms and is a promising management option for PAA. This case report highlights the importance of individualized treatment approaches for rare diseases such as PAA and the need for further research to establish clear guidelines for their management.

## Case presentation

**Figure 1:**
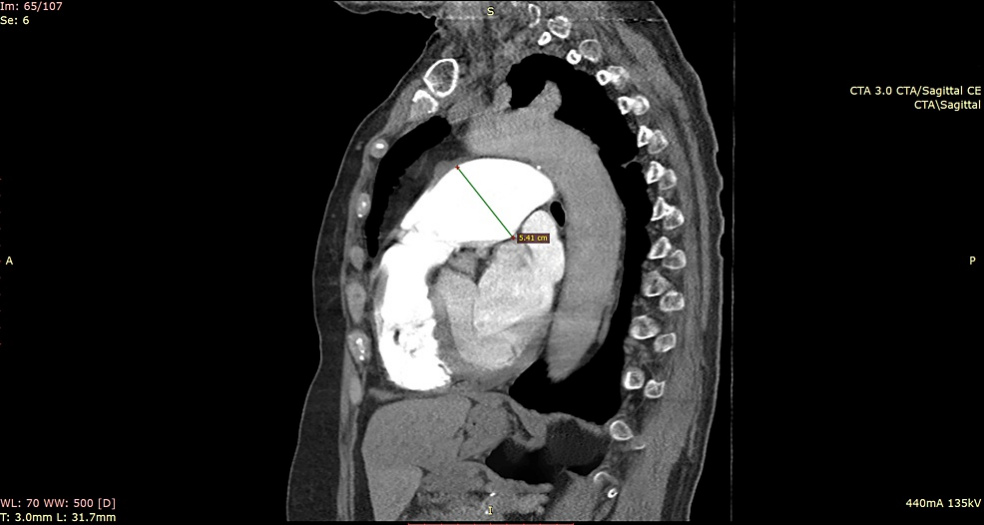
Preoperative computed tomography pulmonary angiogram from October 2020 The angiogram is showing a pulmonary artery aneurysm of 5.4 cm.

An otherwise healthy 68-year-old woman presented on the first of August 2019 with symptoms of persistent palpitations. She was diagnosed with atrioventricular nodal re-entry tachycardia (AVNRT) and treated with metoprolol and propafenone. The patient has a medical history of a benign breast tumor which was resected at the age of 38, as well as a history of four miscarriages. On further examinations, an echocardiogram indicated low degree mitral, aortic, and tricuspid valve regurgitation and a computed tomography angiography (CTA) revealed a PAA of the main PA measuring 5.5 cm x 4.7 cm and mild centrilobular emphysema (see Table 1). The patient denied any dyspnea, chest pain, hoarseness, or syncopal episodes. Eleven months later, the patient was diagnosed with IPAH of 60 mmHg and treated with ramipril and furosemide. A follow-up computed tomography pulmonary angiogram (CTPA) showed a PAA size of 5.4 x 4.9 cm (see Figure 1). The timeline and progression of the patient’s presentation are documented in Table 1.

**Table 1:** Timeline of patient investigations CT: Computed tomography; CTPA: Computed tomography pulmonary angiogram; DLC/O: Diffusing capacity for carbon monoxide; EDV: End-diastolic volume; EF: Ejection fraction; FEV: Forced expiratory volume; FVC: Forced vital capacity; KCO: Carbon monoxide transfer coefficient; LV: Left ventricle; MRI: Magnetic resonance imaging; PAA: Pulmonary artery aneurysm; PG: Pressure gradient; RSVP: Right ventricular systolic pressure; RV: Right ventricle; TR: Tricuspid Regurgitation

Dates	October 2019	November 2019	March 2020	September 2020	October 2020	November 2021	April 2022	January 2023
Investigation	CT angiography	Echocardiogram	Spirometry	Echocardiogram	CTPA	Cardiac MRI	Post-COVID cardiac MRI	Preoperative Cardiac catheterization
Findings	Main PAA measuring 5.5x4.7 cm; Centrilobular pulmonary emphysema	TR max PG= 43.4 mmHg; RSVP=48.4 mmHg; Diagnosis of pulmonary hypertension	FVC 110%; FEV 97%; FEV/FVC= 74%; DLCO 48% KCO 70%	Echo: RVSP 60mmHg	PAA size 5.4x4.9 cm	PAA size 5.8x5.1cm increase of 0.4mm in one year	Depressed LV contractility with index EDV= 117ml/m^2^ , EF=41% (previously EF=57%); Mildly depressed contractility of RV with index EDV= 74ml/m^2^, EF= 46% (previous reports EF was 52%)	Major coronaries are free of disease, and preserved LV systolic function; Severe mitral valve regurgitation and moderate aortic valve regurgitation; Severe dilatation of pulmonary artery trunk

**Figure 2:**
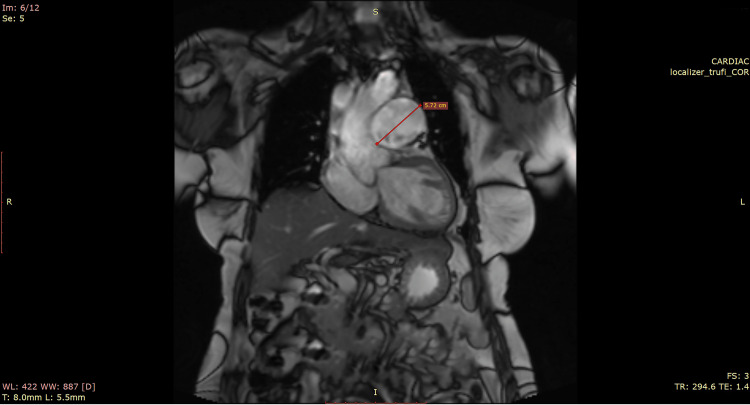
Preoperative cardiac magnetic resonance imaging from November 2021 The image shows a pulmonary artery aneurysm of 5.8 cm.

On 15 October 2021, the patient presented to us to evaluate the need for surgery. Cardiac magnetic resonance imaging (MRI) was performed which demonstrated dilation of the main PA of 5.8 cm x 5.1 cm, with a 4 mm increase in diameter in one year indicating the need for surgery (see Figure 2). However, the patient contracted SARS-CoV-2 and soon after recovery, sustained a leg injury and wound infection, further delaying the surgery. A preoperative exam revealed severe mitral and moderate aortic regurgitation, with preserved left ventricular function. On 11 January 2023, the patient underwent surgery to treat the PAA and repair the valve regurgitations.

Procedure

**Figure 3:**
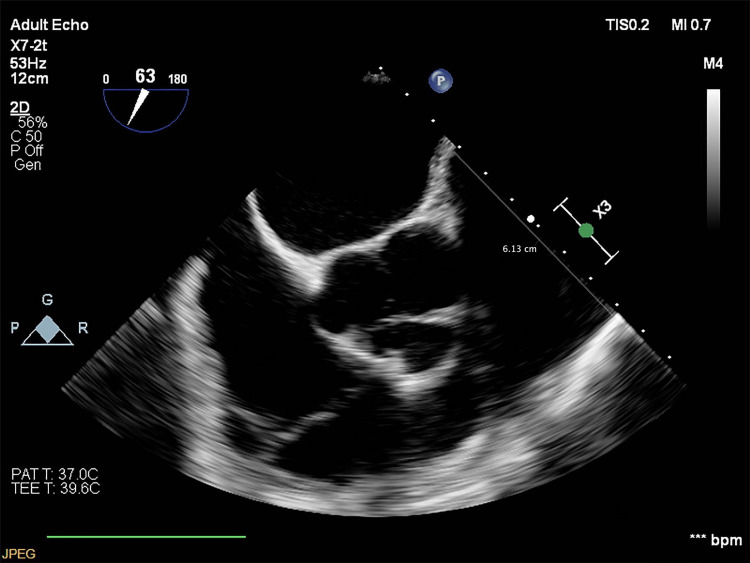
Perioperative transesophageal echocardiography from January 2023 Echocardiography indicates a pulmonary artery aneurysm measuring 6.1 cm.

Perioperative transesophageal echocardiography (TEE) was performed and revealed severe eccentric mitral regurgitation, moderate aortic regurgitation, preserved left ventricular ejection fraction (LVEF), and mild dilation of the left atrium (LA). Furthermore, a mild right ventricular (RV) impairment and mild tricuspid regurgitation was noted, along with a 6.1 cm PAA, and mild pulmonary valve regurgitation (see Figure 3). No shunts were found between the atria or ventricles. 

The patient underwent a median sternotomy procedure, and invasive measurement confirmed the echocardiographic findings. The patient was given systemic heparinization to maintain activated clotting time (ACT) at over 400 seconds. During the procedure, the ascending aorta was cannulated for arterial access, and the vena cava was cannulated using two straight 32F cannulas. Aortic cross-clamping and cardioplegic arrest were performed using 20 ml/kg Del Nido solution through both coronary ostia. The mitral valve was accessed through a left atriotomy and replaced with a 29 mm bioprosthesis. The aortic valve, found to be tricuspid in morphology and structurally altered, was also excised, and replaced with a 21 mm bioprosthesis. 

**Figure 4:**
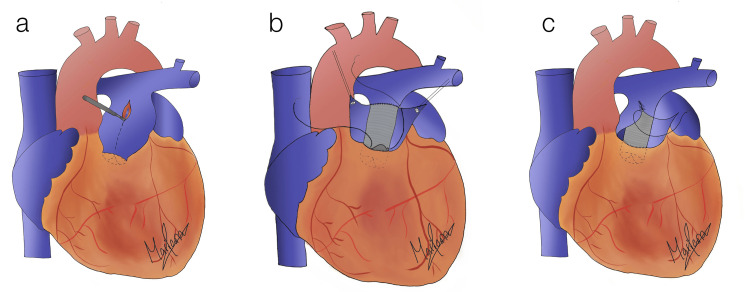
Surgery illustrations (a) Longitudinal incision of the pulmonary artery (PA). Care is taken to visualize the pulmonary valve (PV) and avoid extending up the incision into the PV annulus. The cranial incision was extended to visualize the left and right PAs. (b) Anastomosis of 30mm Dacron prosthetic caudally 1-2 cm above the PV. Cranial anastomosis is performed with care ensuring the right and left PAs are unobstructed. Anastomosis is done using continuous 3-0 prolene sutures. (c) Enclosing the graft with pulmonary vessel walls using a continuous suturing technique.

For the PAA, two prolene stay sutures were placed on the PA and a longitudinal incision was made on the aneurysm between the sutures, carefully extending from 1 cm above the pulmonary valve to the level of the pulmonary bifurcation (see Figure 4a). The pulmonary valve (PV) appeared structurally and morphologically intact. A 30 mm Dacron graft was selected and caudally anastomosed to the PA 1 cm above the PV using continuous 3-0 prolene suture (see Figure 4b). The cranial anastomosis of the graft was performed 1 cm below the pulmonary bifurcation, ensuring no obstruction of either the right or left pulmonary arteries (see Figure 4b). The surrounding tissue was wrapped around the graft using a continuous 3-0 prolene suture (see Figure 4c). 

Following the procedure, echocardiography and TEE were performed to confirm the proper positioning and functioning of the mitral and aortic valve prostheses. The TEE showed an intact pulmonary valve, unchanged RV function, and mild tricuspid valve regurgitation. Hemostasis was achieved and the effects of heparin were reversed using protamine. 

**Figure 5:**
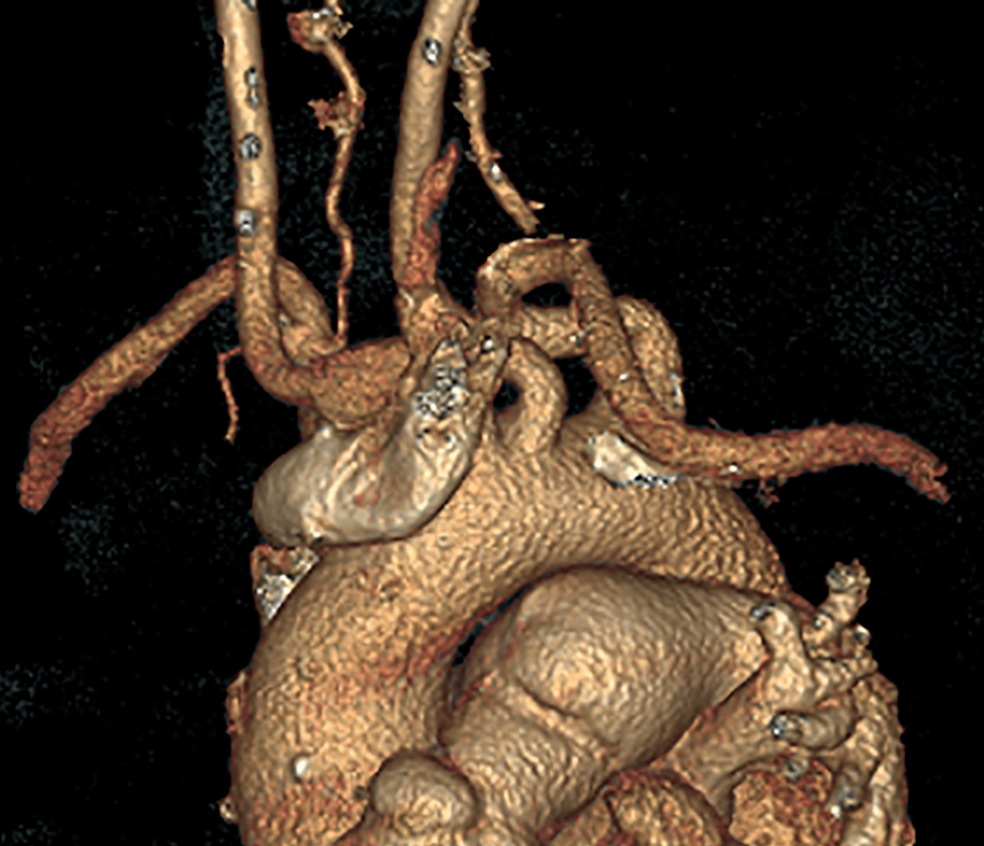
Postoperative computed tomography reconstruction after pulmonary artery aneurysm repair with Dacron graft inclusion technique

Postoperatively, the patient was extubated the following morning and was gradually weaned from inotropic support. Non-invasive ventilatory support was required in the early postoperative period. An upper respiratory tract infection was treated with a targeted antibiotic therapy. The patient made a slow but steady recovery and was discharged to a rehabilitation center after 11 days. The discharge TTE revealed no relevant abnormalities, with well seated, well-functioning aortic and mitral valve prostheses and no relevant tricuspid or pulmonary valve regurgitation. Follow up computed tomography and MRI performed 40 days after discharge showed excellent postoperative results (see Figure 5).

## Discussion

PAA is a rare disease with potentially fatal complications including aneurysm rupture and dissection with cardiac tamponade [3]. Several factors, such as chronic PAH, a PA pressure exceeding 50 mmHg, a PA diameter greater than 75 mm, and a PAA growth rate of over 2 mm per year, have been known to increase the risk of aneurysm rupture [9]. Thus, various treatment options have been used to reduce the risk of these life-threatening complications, including addressing the underlying cause, continual monitoring through imaging tests, and, in severe cases, aggressive surgical intervention [1]. However, due to the rarity of cases, there are currently no standard guidelines for its treatment and each case must be evaluated on an individual basis [1].

In this study, we present the case of a patient with IPAH and a large PAA, measuring 6.1 cm diameter perioperatively. While medical management was initially initiated, Boerrigter et al. notes that progressive dilation of the PA is independent of the pulmonary hemodynamics, i.e., changes in PA pressure and cardiac output, with intrinsic vessel properties more likely to be the cause [10]. In addition, Veldtman et al. reported that the timing of surgical intervention should be determined by changes in RV size and function resulting from PV regurgitation or stenosis and not the size of the aneurysm [11]. Contrastingly, Kreibich et al. proposed the following criteria as indications for surgery: an absolute PAA diameter ≥ 5.5 cm; an increase in diameter of ≥ 0.5 cm in six months; compression of adjacent structures; thrombus formation in the aneurysm sack; appearance of clinical symptoms; evidence of valvular pathologies or shunt flow; verification of PAH; or signs of rupture or dissection [5]. The patient experienced an increase in PAA diameter of 4 mm over the course of a year, as well as mild RV impairment and PV regurgitation, thus fulfilling both the proposed indications which prompted concern and required surgical repair. 

In our case, the patient presented with a high-risk PAA of the pulmonary trunk. According to research, aneurysmorrhaphy [12,13] and aneurysmectomy [14,15] are well-described surgeries for the treatment of PA aneurysms confined to the pulmonary trunk. While aneurysmorrhaphy is a straightforward and quick surgical option, it only reduces the diameter of the PA without addressing the underlying abnormality in the vessel wall [11]. Since the aneurysmal wall is not removed, this technique may lead to recurrent dilatation, especially in cases of associated PAH [11]. On the other hand, aneurysmectomy involves total excision of the aneurysm along with a graft-interposition technique. However, one adverse effect of using graft-interposition techniques is the formation of a pseudoaneurysm due to the partial dehiscence from the suture lines [16,17].

The graft inclusion technique described in this case involves a graft insertion, and subsequent enclosure of the graft by the remnant of the disease pulmonary artery, resulting in a potential space between the graft and the vessel wall. The benefits of this technique have been previously studied for the treatment of aortic aneurysms [18]. Although the inclusion technique also presents a risk of development of pseudoaneurysms, the clinical implications are less severe as the vessel wall jacket restricts the blood flow [18]. Moreover, pseudoaneurysms that are not protected by a vessel wall present a more ominous picture and have been found to have a higher likelihood of anastomotic infection [17,18]. Thus, the use of the graft inclusion technique for the repair of a main PAA may be beneficial in addressing and eliminating the complications and adverse effects of the conventional surgical interventions. However, it should be noted that regardless of the technique being used, continued follow-up should be pursued to document the stability of the repair.

## Conclusions

In this report, we present a case of successful large PAA repair in a patient with concomitant PAH. To the best of our knowledge, this is the first reported case of a PAA being repaired using the graft inclusion technique. The use of the graft inclusion technique proved to be a rapid and straightforward method for repairing the PAA with effective hemostasis, as demonstrated by the postoperative TEE. However, further research with a larger sample size and direct comparison of the different surgical interventions to the use of the inclusion technique should be performed with longer follow-ups to evaluate the safety and efficacy of this approach.
